# Association between advanced lung cancer inflammation index levels and ischemic stroke in patients with atrial fibrillation: a propensity score-matching analysis

**DOI:** 10.3389/fneur.2025.1652042

**Published:** 2026-01-07

**Authors:** Ying Li, Xuelin Lu, Zuoan Qin, Jiangbiao Yu

**Affiliations:** 1 Department of Science and Education, Changde Hospital, Xiangya School of Medicine, Central South University (The First People’s Hospital of Changde City), Changde, China; 2 Department of Pathology, Changde Hospital, Xiangya School of Medicine, Central South University (The First People’s Hospital of Changde City), Changde, China; 3 Department of Cardiology, Changde Hospital, Xiangya School of Medicine, Central South University (The First People’s Hospital of Changde City), Changde, China

**Keywords:** ALI, atrial fibrillation, ischemic stroke, inflammation, propensity score matching

## Abstract

**Introduction:**

Inflammation is closely associated with atrial fibrillation (AF) complicated by stroke. The Advanced Lung Cancer Index (ALI) is a comprehensive indicator of inflammation; however, its relationship with AF-related stroke is unclear. Therefore, this retrospective study was conducted to explore the correlation between ALI and ischemic stroke in patients with AF.

**Methods:**

Patients were divided into two groups according to the optimal cutoff value of ALI: low ALI and high ALI groups. The primary outcome was ischemic stroke in patients with AF. To ensure robustness of the findings, propensity score matching, multivariate logistic regression, inverse probability weighting models, and doubly robust analysis were performed.

**Results:**

Of 2,630 eligible patients (screened: 1,879), 15.6% had a past medical history of ischemic stroke. Restricted cubic splines showed a linear dose–response relationship between baseline ALI and stroke risk (p for non-linearity = 0.46). A propensity-adjusted doubly robust analysis of 874 matched patients revealed a graded protective effect with increasing ALI quintiles: compared with the Q1 group, the Q4 and Q5 groups had odds ratios of 0.38 (95% CI 0.25-0.58) and 0.54 (0.35-0.82), respectively. Consistency across subgroups and sensitivity analysis confirmed the robustness of the results.

**Conclusion:**

ALI showed a significant protective association with ischemic stroke in participants with AF, as increased ALI level was associated with lower prevalence of ischemic stroke.

## Introduction

Atrial fibrillation (AF) is one of the most common persistent arrhythmias worldwide, with both its prevalence and incidence constantly increasing (1, 2). China is a populous country with a large number of patients with AF, which has exceeded 30 million (3). AF poses a great threat, which can lead to complications such as ischemic stroke, heart failure, myocardial infarction, renal function impairment, and cognitive decline, all of which severely affect the quality of life of patients and place a heavy burden on society and families (4–7). AF can lead to an increase in ischemic stroke risk by five times and mortality rate by two times (8). Research has shown that over 20% of ischemic stroke cases are related to AF (9), and the prognosis of AF after a stroke event is poor. Therefore, preventing stroke events in patients with AF has become a key part of the clinical treatment strategy, with stroke risk assessment being an important step in effective prevention. Currently, clinical practice guidelines recommend the CHA2DS2-VASc score as an important tool for assessing stroke risk in non-valvular AF (10). However, in real-life clinical practice, this tool has been found to have certain limitations, as it only includes clinical indicators such as age and comorbidities (hypertension, diabetes, heart failure, etc.), while overlooking indicators such as the duration of AF, cardiac morphology and function, and blood biochemistry. Incorporating new predictors may help optimize the current scoring system for stroke risk in patients with AF. Therefore, identifying these new biomarkers has become the future direction for the prevention and treatment of stroke in these patients (11). Studies have shown that inflammation is involved in the initiation and maintenance of AF (12), and that it is related to the occurrence of ischemic stroke in patients with AF (13, 14). The advanced lung cancer inflammation index (ALI) is a comprehensive assessment that reflects the complex interaction between systemic inflammation, immune function, and nutritional status. As a comprehensive indicator of inflammation, it integrates three key parameters: body mass index (BMI), plasma albumin (Alb) level, and neutrophil-to-lymphocyte ratio (NLR). In recent years, multiple studies have shown that ALI can also serve as a prognostic indicator for other diseases, such as multiple myeloma, Crohn’s inflammatory bowel disease, and coronary heart disease (15–17). The importance of optimizing the previous stroke risk assessment model and identifying new biomarkers in patients with AF has been suggested; however, no studies have been conducted on the correlation between ALI and AF combined with ischemic stroke. Therefore, this retrospective study was conducted to explore the correlation between ALI and ischemic stroke in patients with AF and to analyze its predictive value for assessing stroke risk in these patients.

## Materials and methods

### Data collection

This cross-sectional retrospective study was conducted using medical records extracted from the Hospital Information System database, which contains comprehensive information on patients admitted to our institution between January 1, 2022, and December 31, 2024, including 2,630 adult patients admitted to the hospital for AF. This study was conducted in accordance with the Reporting of Observational Studies in Epidemiology guidelines (18).

### Study population

Inclusion criterion: patients aged over 18 years hospitalized with AF, including paroxysmal or persistent AF. Exclusion criteria: (1) patients with valvular heart disease, congestive heart failure or cardiomyopathy, left atrial diastolic diameter >60 mm, or cardiac function class IV; (2) patients with myocardial infarction or fatal arrhythmia within 3 months; (3) patients with major diseases of the brain, lungs, liver, kidneys, and other organs; (4) malignant tumors; and (5) hyperthyroidism.

### Measurement of ALI

ALI was calculated as follows: ALI = BMI × Alb/NLR, where BMI = weight in kilograms/height in meters^2^, Alb = serum alb in grams per decaliter, and NLR was derived using the absolute neutrophil count/absolute lymphocyte count (19).

### Identification of ischemic stroke

Ischemic stroke was ascertained a prior history, using neurologist-documented diagnoses from previous clinical encounters. It meet the defined according to the World Health Organization (WHO) diagnostic criteria (ICD-10 Code: I63) as “rapidly developing clinical symptoms or signs of focal, and at times global, loss of cerebral function, with symptoms lasting more than 24 h or leading to death, with no apparent cause other than that of vascular origin (20, 21).” This definition excludes clinical cases of primary cerebral tumors, cerebral metastasis, subdural hematoma, post-seizure palsy, and brain trauma.

### Covariates definitions

Demographic data included age, sex, smoking status, drinking status, and anthropometric measurements (body weight and height) obtained within 24 h of admission. AF was classified into paroxysmal (episodes ≤7 days) and persistent (episodes >7 days) types, and cardiac function was assessed using the New York Heart Association (NYHA) Functional Classification (Grades I–IV). Vital signs included systolic and diastolic blood pressure and heart rate. Comprehensive medical history included cardiovascular comorbidities (heart valve disease, hypertension, diabetes, hyperlipidemia, and coronary heart disease) and surgical interventions (radiofrequency ablation, pacemaker, and coronary stent implantation). Laboratory parameters, obtained after ≥6 h of fasting within 24 h of hospitalization, included an extensive panel of hematological markers (hemoglobin, red blood cell distribution width-coefficient of variation, lymphocyte, neutrophil, monocyte, and platelet counts), biochemical markers (albumin, glucose, globulin, lipid profile, creatinine, ALT, uric acid, and urea), cardiac biomarkers (C-reactive protein [CRP], N-terminal pro B-type natriuretic peptide [NT-proBNP], and cardiac troponin I [CTnI]), and coagulation parameters (prothrombin time [PT], international normalized ratio [INR], and anticoagulant usage), providing a comprehensive clinical profile for analysis.

### Statistical analysis

**Figure 1:**
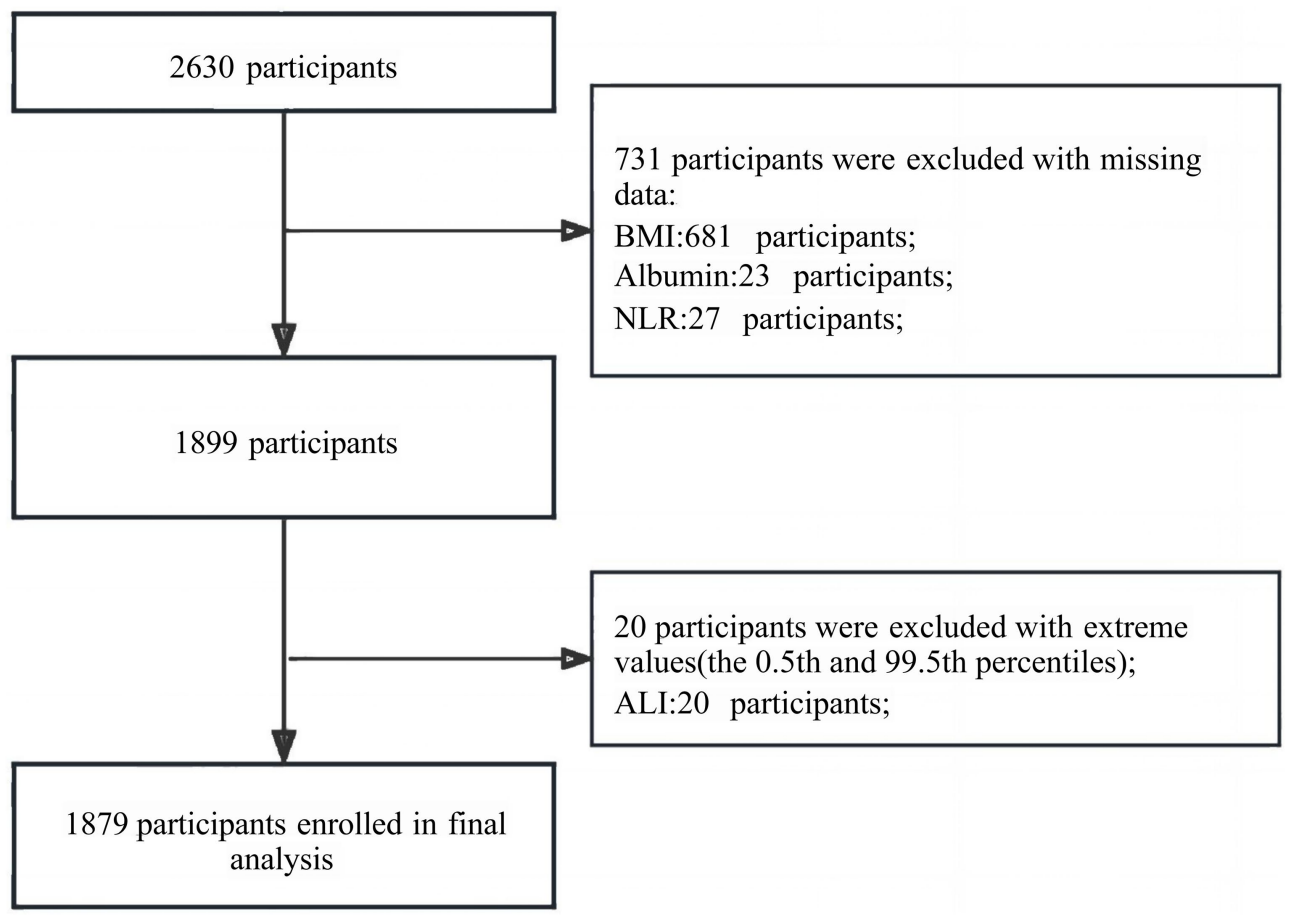
Flow chart of the study population.

Because electronic medical records were used, data were not complete for all variables, as shown in Supplementary Table 1. Missing data for ALI exposure (BMI [*n* = 681], alb [*n* = 23], NLR [*n* = 27]) were deleted, whereas data of the outcome variable, ischemic stroke, were complete and truncated at the 0.5th and 99.5th percentiles of ALI to exclude extreme outliers (*n* = 20). In total, 1879 patients (1,020 males and 859 females) were enrolled in the analysis (Figure 1). To address missing covariate data before statistical analysis, multivariate single imputation was performed to obtain unbiased estimates of the association between ALI and the outcome, using a Bayesian Ridge model as the estimator at each step of the round-robin imputation (22).

Histogram distribution was used for assess the normality of variables. All normally distributed continuous variables were expressed as mean ± standard deviation (SD), and skewed continuous variables were described as median (interquartile range [IQR]), while categorical variables were presented as frequencies (%). A paired *t*-test or Wilcoxon signed-rank test was applied for paired factor differences within groups. Comparison of continuous variables among groups was performed with the use of the independent samples Student’s t-test or Mann–Whitney U-test depending on the normality of the distribution, while the χ^2^ test was used for categorical variables. To investigate the potential nonlinear dose–response relationship between ALI and ischemic stroke, a restricted cubic spline model was employed, which allowed for the development of smooth curves. In this model, the ALI was treated as a continuous variable with four knots positioned at the 5th, 35th, 65th, and 95th percentiles, as recommended by Harrell. Nonlinearity was assessed using a likelihood ratio test that compared a model with only a linear term with a model that included both linear and cubic spline terms. We analyzed ALI both as a continuous variable (natural logarithm transformation) and as a categorical variable (by quintile [Q]: Q1, <14.46; Q2, 14.46–22.48; Q3, 22.48–30.71; Q4, 30.71–43.03; and Q5, >43.03). All predictors with *p* < 0.10 by univariate analysis were retained in the multivariate models. Multivariate logistic regression analysis was performed to investigate the association for ALI and ischemic stroke.

We determined the optimal ALI cutoff thresholds using receiver operating characteristic (ROC) curves with Youden’s index correction (23), then performed propensity score matching (PSM) to adjust for baseline characteristic differences between the high- and low-ALI groups. The covariates included in the PSM analysis encompassed demographic variables (age and gender), lifestyle factors (smoking status and alcohol consumption), clinical measurements (blood pressure, hemoglobin, platelet count, creatinine, uric acid, globulin, and fasting blood glucose), lipid profile parameters (triglycerides, high-density lipoprotein [HDL] cholesterol, and low-density lipoprotein cholesterol), cardiac biomarkers (BNP and CRP), coagulation parameters (PT, INR, and D2 polymer), as well as medical history, operator details, anticoagulant use, types of AF, and NYHA Functional Classification. Participants were matched one-to-one using the nearest neighbor technique, which matched each treated unit to the closest control using caliper width set at 0.1 SD of the logit of the estimated propensity score, without iteration. Baseline comparisons between the covariates were conducted for the matched and unmatched samples. Balance diagnosis was performed using the standardized difference method, which compares the difference in means of each covariate in units of the pooled SD for the matched and unmatched samples. Successful matching is indicated when the absolute standardized differences of means is less than 0.25 (24). Based on the propensity score, the stabilized inverse probability of the treatment weighting (IPTW) was calculated (25).

Four models were used in the multivariable logistic regression analysis: unadjusted, multivariable, propensity score-adjusted multivariable, and doubly robust. Multivariable logistic regression models were adjusted for age, sex, anticoagulant therapy, relevant comorbidities, NY heart function classification and types of atrial fibrillation. The doubly robust approach (26–29) integrated propensity score-based IPTW with outcome regression. Stabilized IPTW weights were used to address confounding factors, with direct adjustments for residual imbalances in Age, sex, anticoagulant therapy, relevant comorbidities, NY heart function classification and types of atrial fibrillation within the weighted logistic regression.

Within the matched patient group, we assessed heterogeneity of treatment effects with tests of interaction and subgroup analyses, exploring the effect of age, use of anticoagulants, smoking status, drinking status, surgical history, hypertension, and diabetes. A forest plot was generated. Owing to limited reports on ALI level in patients with AF and the unclear association between ALI and decreased AF-related ischemic stroke, we conducted an E-value analysis in the matched patient group to investigate the potential effects of this unmeasured confounder on our results (30, 31).

All analyses were performed using R Statistical Software (Version 4.2.2, http://www.R-project.org, The R Foundation) and the Free Statistics Analysis Platform (Version 1.9, Beijing, China, http://www.clinicalscientists.cn/freestatistics). A two-sided *p* value < 0.05 was considered statistically significant.

## Results

### Characteristics of the patients

A total of 1,879 patients were included in this study after a rigorous screening process in accordance with predetermined inclusion and exclusion criteria. Among these patients, the overall prevalence of ischemic stroke was 15.6% (*n* = 295).

The study population was categorized into high ALI group and low ALI group according to the optimal cut-off value. In comparing both groups, those in the low ALI group were significantly older (median 73.0 [IQR 67.0–79.0] vs. 70.0 [63.0–75.5], *p* < 0.001) and had a higher heart rate (84.0 [72.0–97.0] vs. 81.0 [69.5–94.0], *p* = 0.007). On the other hand, those in the high ALI group had a higher prevalence of coronary heart disease (35.7% vs. 29.6%, *p* = 0.012) but a lower prevalence of hyperlipidemia (16.1% vs. 20.4%, *p* = 0.034), alongside a markedly lower utilization rate of radiofrequency ablation (6.4% vs. 12.4%, *p* < 0.001). Clinically significant disparities were observed in hemoglobin level (115.1 ± 23.1 vs. 126.7 ± 21.0 g/L, p < 0.001),white blood cell count (7.6 ± 4.4 vs. 5.8 ± 2.4 × 10^9^/L, *p* < 0.001),while Group 2 had a higher HDL cholesterol level (1.0 ± 0.3 vs. 1.1 ± 0.4 mmol/L, *p* = 0.030), leukocyte count (7.6 ± 4.4 vs. 5.8 ± 2.4 × 10^9^/L, *p* < 0.001), and inflammatory marker level (NT-proBNP: 4,968.5 ± 7,270.5 vs. 2,500.1 ± 4,399.7 pg./mL; CRP: 17.0 ± 26.4 vs. 7.1 ± 15.9 mg/L, both *p* < 0.001). Electrolyte imbalances were noted in sodium (139.4 ± 4.5 vs. 140.5 ± 3.2 mmol/L) and chloride (105.0 ± 5.7 vs. 106.0 ± 3.8 mmol/L, both *p* < 0.001) levels. No significant differences were observed in anticoagulant regimens (*p* = 0.929) or sex distribution (*p* = 0.122). (Table 1).

**Table 1:** General characteristics of the study population according to ALI before and after propensity score matching (PSM).

Variables	Before PSM					After PSM				
	Total (*n* = 1879)	Low level group (*n* = 516)	High level group (*n* = 1,363)	*p*	Statistic	Total (*n* = 722)	Low level group (*n* = 361)	High level group (*n* = 361)	*p*	Statistic
Gender, *n* (%)				0.122	2.388				0.822	0.051
Male	859 (45.7)	221 (42.8)	638 (46.8)			319 (44.2)	158 (43.8)	161 (44.6)		
Female	1,020 (54.3)	295 (57.2)	725 (53.2)			403 (55.8)	203 (56.2)	200 (55.4)		
Smoking status, *n* (%)				0.529	0.396				0.691	0.158
No	1,277 (68.0)	345 (66.9)	932 (68.4)			487 (67.5)	241 (66.8)	246 (68.1)		
Yes	602 (32.0)	171 (33.1)	431 (31.6)			235 (32.5)	120 (33.2)	115 (31.9)		
Alcohol status, *n* (%)				0.26	1.271				0.895	0.017
No	1743 (92.8)	473 (91.7)	1,270 (93.2)			659 (91.3)	329 (91.1)	330 (91.4)		
Yes	136 (7.2)	43 (8.3)	93 (6.8)			63 (8.7)	32 (8.9)	31 (8.6)		
Heart rate, bpm, Median (IQR)	82.0 (70.0, 95.0)	84.0 (72.0, 97.0)	81.0 (69.5, 94.0)	0.007	7.312	83.0 (71.0, 96.0)	83.0 (70.0, 96.0)	83.0 (72.0, 97.0)	0.934	0.007
Heart valve disease, *n* (%)				0.182	1.778				0.14	2.177
No	1,561 (83.1)	419 (81.2)	1,142 (83.8)			597 (82.7)	291 (80.6)	306 (84.8)		
Yes	318 (16.9)	97 (18.8)	221 (16.2)			125 (17.3)	70 (19.4)	55 (15.2)		
Diabetes, *n* (%)				0.661	0.192				0.385	0.755
No	1,523 (81.1)	422 (81.8)	1,101 (80.9)			591 (81.9)	291 (80.6)	300 (83.1)		
Yes	354 (18.9)	94 (18.2)	260 (19.1)			131 (18.1)	70 (19.4)	61 (16.9)		
Hyperlipidemia, *n* (%)				0.034	4.482				0.385	0.756
No	1,518 (80.8)	433 (83.9)	1,085 (79.6)			624 (86.4)	308 (85.3)	316 (87.5)		
Yes	361 (19.2)	83 (16.1)	278 (20.4)			98 (13.6)	53 (14.7)	45 (12.5)		
Coronary heart disease, *n* (%)				0.012	6.306				0.248	1.337
No	1,291 (68.7)	332 (64.3)	959 (70.4)			455 (63.0)	235 (65.1)	220 (60.9)		
Yes	588 (31.3)	184 (35.7)	404 (29.6)			267 (37.0)	126 (34.9)	141 (39.1)		
Radiofrequency ablation, *n* (%)				< 0.001	14.062				0.55	0.357
No	1,677 (89.2)	483 (93.6)	1,194 (87.6)			674 (93.4)	335 (92.8)	339 (93.9)		
Yes	202 (10.8)	33 (6.4)	169 (12.4)			48 (6.6)	26 (7.2)	22 (6.1)		
Pacemaker implantation surgery, *n* (%)				0.553	0.353				0.482	0.494
No	1800 (95.8)	492 (95.3)	1,308 (96)			688 (95.3)	346 (95.8)	342 (94.7)		
Yes	79 (4.2)	24 (4.7)	55 (4)			34 (4.7)	15 (4.2)	19 (5.3)		
Hemoglobin, g/L, Mean ± SD	123.5 ± 22.2	115.1 ± 23.1	126.7 ± 21.0	< 0.001	106.118	118.8 ± 22.7	119.3 ± 21.2	118.2 ± 24.1	0.497	0.462
Platelet, 10⁹/L, Mean ± SD	165.0 ± 62.4	164.8 ± 65.0	165.1 ± 61.4	0.914	0.012	161.5 ± 58.8	160.6 ± 60.6	162.4 ± 57.1	0.682	0.168
RDW SD, %, Mean ± SD	46.3 ± 6.3	47.6 ± 6.4	45.8 ± 6.2	< 0.001	29.992	47.3 ± 6.1	47.1 ± 6.1	47.5 ± 6.2	0.423	0.643
White blood cell, 10⁹/L, Mean ± SD	6.3 ± 3.2	7.6 ± 4.4	5.8 ± 2.4	< 0.001	122.149	6.5 ± 2.3	6.5 ± 2.5	6.5 ± 2.1	0.82	0.052
Red blood cell, 10^12^/L, Mean ± SD	4.2 ± 2.7	3.9 ± 0.8	4.3 ± 3.1	< 0.001	10.985	4.0 ± 0.7	4.0 ± 0.7	4.0 ± 0.8	0.996	0
Blood potassium, mmol/L, Mean ± SD	3.9 ± 0.5	4.0 ± 0.6	3.9 ± 0.5	0.036	4.424	4.0 ± 0.5	4.0 ± 0.6	4.0 ± 0.5	0.53	0.395
Blood sodium, mmol/L, Mean ± SD	140.2 ± 3.6	139.4 ± 4.5	140.5 ± 3.2	< 0.001	35.922	139.8 ± 3.9	139.9 ± 4.4	139.8 ± 3.4	0.754	0.098
Blood chlorine, mmol/L, Mean ± SD	105.7 ± 4.4	105.0 ± 5.7	106.0 ± 3.8	< 0.001	18.286	105.4 ± 5.0	105.6 ± 5.8	105.1 ± 4.2	0.204	1.614
Blood calcium, mmol/L, Mean ± SD	2.3 ± 1.3	2.3 ± 1.4	2.3 ± 1.3	0.561	0.339	2.2 ± 0.2	2.2 ± 0.2	2.2 ± 0.3	0.04	4.248
Creatinine, μmol/L, Mean ± SD	85.3 ± 86.2	106.2 ± 113.9	77.4 ± 71.6	< 0.001	42.519	96.0 ± 113.7	97.3 ± 108.9	94.8 ± 118.5	0.77	0.086
Uric acid, μmol/L, Mean ± SD	382.1 ± 122.4	393.0 ± 139.3	378.0 ± 115.1	0.017	5.672	381.7 ± 125.6	383.3 ± 125.2	380.0 ± 126.2	0.725	0.124
Globulin, g/L, Mean ± SD	25.1 ± 5.2	25.7 ± 5.4	24.9 ± 5.1	< 0.001	10.957	25.6 ± 5.2	25.1 ± 5.2	26.1 ± 5.0	0.009	6.919
Fasting blood glucose, Mean ± SD	5.7 ± 2.1	5.8 ± 2.6	5.7 ± 1.8	0.166	1.922	5.8 ± 2.1	5.7 ± 2.1	5.9 ± 2.0	0.319	0.993
Triglyceride, mmol/L, Mean ± SD	1.5 ± 3.9	1.2 ± 0.8	1.6 ± 4.6	0.095	2.798	1.2 ± 0.7	1.2 ± 0.8	1.2 ± 0.7	0.694	0.155
HDL cholesterol, mmol/L, Mean ± SD	1.1 ± 0.4	1.0 ± 0.4	1.1 ± 0.4	0.226	1.466	1.1 ± 0.4	1.1 ± 0.4	1.1 ± 0.4	0.148	2.098
LDL cholesterol, mmol/L, Mean ± SD	2.1 ± 0.9	2.0 ± 1.0	2.2 ± 0.9	0.005	7.897	2.1 ± 0.9	2.0 ± 1.0	2.1 ± 0.8	0.46	0.548
BNP, ng/L, Mean ± SD	3192.8 ± 5474.1	4968.5 ± 7270.5	2500.1 ± 4399.7	< 0.001	76.838	4097.2 ± 6391.5	4114.3 ± 6170.4	4080.1 ± 6613.7	0.943	0.005
CRP, mg/L, Mean ± SD	9.9 ± 20.0	17.0 ± 26.4	7.1 ± 15.9	< 0.001	93.139	11.7 ± 21.5	10.8 ± 18.9	12.5 ± 23.9	0.293	1.109
PT, sec, Mean ± SD	13.5 ± 4.5	13.3 ± 3.5	13.5 ± 4.9	0.427	0.63	13.2 ± 3.7	13.4 ± 3.6	13.0 ± 3.7	0.103	2.668
INR, Mean ± SD	1.2 ± 0.8	1.2 ± 0.6	1.2 ± 0.9	0.349	0.878	1.2 ± 0.6	1.2 ± 0.7	1.1 ± 0.4	0.121	2.412
D2 polymer, mg/L, Mean ± SD	1.2 ± 2.3	1.3 ± 1.8	1.2 ± 2.5	0.699	0.149	1.1 ± 1.5	1.1 ± 1.7	1.0 ± 1.4	0.179	1.812
Anticoagulants, *n* (%)				0.929	Fisher				0.681	1.506
0	559 (29.7)	152 (29.5)	407 (29.9)			206 (28.5)	97 (26.9)	109 (30.2)		
1	1,114 (59.3)	306 (59.3)	808 (59.3)			437 (60.5)	223 (61.8)	214 (59.3)		
2	148 (7.9)	41 (7.9)	107 (7.9)			55 (7.6)	30 (8.3)	25 (6.9)		
3	55 (2.9)	17 (3.3)	38 (2.8)			24 (3.3)	11 (3)	13 (3.6)		
4	3 (0.2)	0 (0)	3 (0.2)							
Age, yrs., Median (IQR)	71.0 (64.0, 77.0)	73.0 (67.0, 79.0)	70.0 (63.0, 75.5)	< 0.001	54.063	73.0 (67.0, 79.0)	73.0 (67.0, 79.0)	73.0 (67.0, 79.0)	0.415	0.665
Systolic pressure, Median (IQR)	126.0 (113.0, 141.0)	125.0 (112.0, 140.0)	127.0 (114.0, 142.0)	0.153	2.042	126.0 (114.0, 142.0)	126.0 (113.0, 140.0)	126.0 (115.0, 143.0)	0.485	0.487
Diastolic pressure, Median (IQR)	78.0 (71.0, 88.0)	77.0 (70.0, 86.0)	79.0 (71.0, 88.0)	0.004	8.513	78.0 (72.0, 88.0)	78.0 (72.0, 88.0)	79.0 (71.0, 88.0)	0.873	0.026

PSM, propensity score matching; Sex, biological sex classification; yrs, years; HR, heart rate; bpm, beats per minute; SBP, systolic blood pressure; DBP, diastolic blood pressure; HVD, heart valve disease; DM, diabetes mellitus; Hb, hemoglobin; PLT, platelet count; RDW, red cell distribution width; WBC, white blood cell count; RBC, red blood cell count; K⁺, potassium; Na⁺, sodium; Cl⁻, chloride; Ca^2^⁺, calcium; Cr, creatinine; UA, uric acid; Glob, globulin; FBG, fasting blood glucose; TG, triglycerides; HDL-C, high-density lipoprotein cholesterol; LDL-C, low-density lipoprotein cholesterol; BNP, B-type natriuretic peptide; CRP, C-reactive protein; PT, prothrombin time; INR, international normalized ratio; D-dimer, fibrin degradation fragment D; SD, standard deviation; IQR, interquartile range.

To adjust for differences in baseline characteristics between the two groups, we performed 1:1 PSM and evaluated outcomes. All baseline characteristics, except for ischemic stroke, were included in a generalized linear model as categorical factors to generate propensity scores. PSM yielded 722matched patients (Group 1: 361; Group 2: 361). Covariate balance was achieved across all predefined confounders, with standardized mean differences(SMD)reduced from a pre-matching maximum of 0.46 to <0.15 post-matching (Supplementary materials). After PSM, baseline characteristics, including demographics, lifestyle factors, vital signs and laboratory parameters were generally well balanced between the two groups.

### Association between ALI and ischemic stroke

Table 2 presents the crude, multivariable-adjusted, and propensity-weighted ORs for ischemic stroke related to ALI levels. When ALI as a continuous variable (per log scale), each unit increase was associated with reduced ischemic stroke prevalence: crude OR = 0.75 (95% CI: 0.62–0.9); multivariable adjusted OR = 0.74 (95% CI: 0.61–0.89); IPTW OR = 0.74 (95% CI: 0.61–0.9); and doubly robust OR = 0.74 (95% CI: 0.61–0.9), respectively (*p* < 0.05). Meanwhile, further adjustment did not significantly affect the results. Similarly, when categorized into quintiles, this association remained statistically significantly negative, as the OR for Q5 compared with Q1 was 0.54 (95% CI: 0.35–0.82), and the OR for Q4 compared with Q1 was 0.48 (95% CI: 0.32–0.72), even after controlling for potential confounders. In the propensity score-weighted model, the OR for Q4 and Q5 were 0.37 (95% CI: 0.24–0.56) and 0.51 (95% CI: 0.34–0.77), respectively (*p* < 0.05). In doubly robust regression analysis incorporating IPTW and outcome model adjustment, statistically significant inverse associations were observed between higher ALI quartiles and ischemic stroke. Compared with Q1 (reference), the adjusted ORs were 0.38 (95% CI: 0.25–0.58) for Q4 and 0.54 (95% CI: 0.35–0.82) for Q5 (P-trend<0.001). Although the OR of Q5 was slightly higher than that of Q4, both were significantly lower than those of Q1 and Q2, and the trend direction was consistent.

**Table 2:** The association between ALI and ischemic stroke.

Variable	n.total	n.event_%	Crude model	*P*	Multivariable model	P	IPTW	*P*	Doubly robust model	P
log ALI	1879	292 (15.5)	0.75 (0.62~0.9)	0.002	0.75 (0.63~0.91)	0.003	0.74 (0.61~0.89)	0.002	0.74 (0.61~0.9)	0.003
ALI groups										
Low level group	516	106 (20.5)	1(Ref)		1(Ref)		1(Ref)		1(Ref)	
High level group	1,363	187 (13.7)	0.62 (0.47~0.8)	<0.001	0.65 (0.5~0.85)	0.002	0.66 (0.51~0.86)	0.002	0.66 (0.51~0.87)	0.003
ALI by tertile										
Q1(2.38~19.38)	623	124 (19.9)	1(Ref)		1(Ref)		1(Ref)		1(Ref)	
Q2(19.39~34.56)	633	95 (15)	0.71 (0.53~0.95)	0.023	0.69 (0.51~0.93)	0.015	0.8 (0.6~1.07)	0.13	0.79 (0.59~1.06)	0.115
Q3(34.57~103.01)	623	73 (11.7)	0.53 (0.39~0.73)	<0.001	0.55 (0.4~0.76)	<0.001	0.5 (0.36~0.69)	<0.001	0.52 (0.38~0.73)	<0.001
Trend.test	1879	292 (15.5)	0.73 (0.62~0.85)	<0.001	0.74 (0.63~0.87)	<0.001	0.72 (0.61~0.84)	<0.001	0.73 (0.62~0.86)	<0.001
ALI by quintiles										
Q1(2.38~14.46)	370	75 (20.3)	1(Ref)		1(Ref)		1(Ref)		1(Ref)	
Q2(14.47~22.48)	380	70 (18.4)	0.89 (0.62~1.28)	0.522	0.88 (0.61~1.27)	0.504	0.7 (0.49~1.01)	0.056	0.71 (0.49~1.02)	0.063
Q3(22.49~30.71)	379	60 (15.8)	0.74 (0.51~1.08)	0.115	0.7 (0.48~1.02)	0.063	0.82 (0.57~1.18)	0.282	0.79 (0.55~1.13)	0.2
Q4(30.72~43.04)	380	41 (10.8)	0.48 (0.32~0.72)	<0.001	0.48 (0.32~0.73)	0.001	0.37 (0.24~0.56)	<0.001	0.38 (0.25~0.58)	<0.001
Q5(43.05~103.01)	370	46 (12.4)	0.56 (0.37~0.83)	0.004	0.58 (0.38~0.87)	0.008	0.51 (0.34~0.77)	0.001	0.54 (0.35~0.82)	0.004
Trend.test	1879	292 (15.5)	0.84 (0.76~0.91)	<0.001	0.84 (0.77~0.92)	<0.001	0.82 (0.75~0.9)	<0.001	0.83 (0.75~0.91)	<0.001

Adjusted for: Age, sex, anticoagulant therapy, relevant comorbidities, NY heart function classification, and types of atrial fibrillation.

In addition, restricted cubic spline (RCS) of the association between ALI and ischemic stroke is shown in Supplementary material. ALI levels and the incidence of ischemic stroke had a negative association when all potential confounders were taken into account (nonlinearity: *p* = 0.461) (Supplementary Figure 1).

### Subgroup analyses

**Figure 2:**
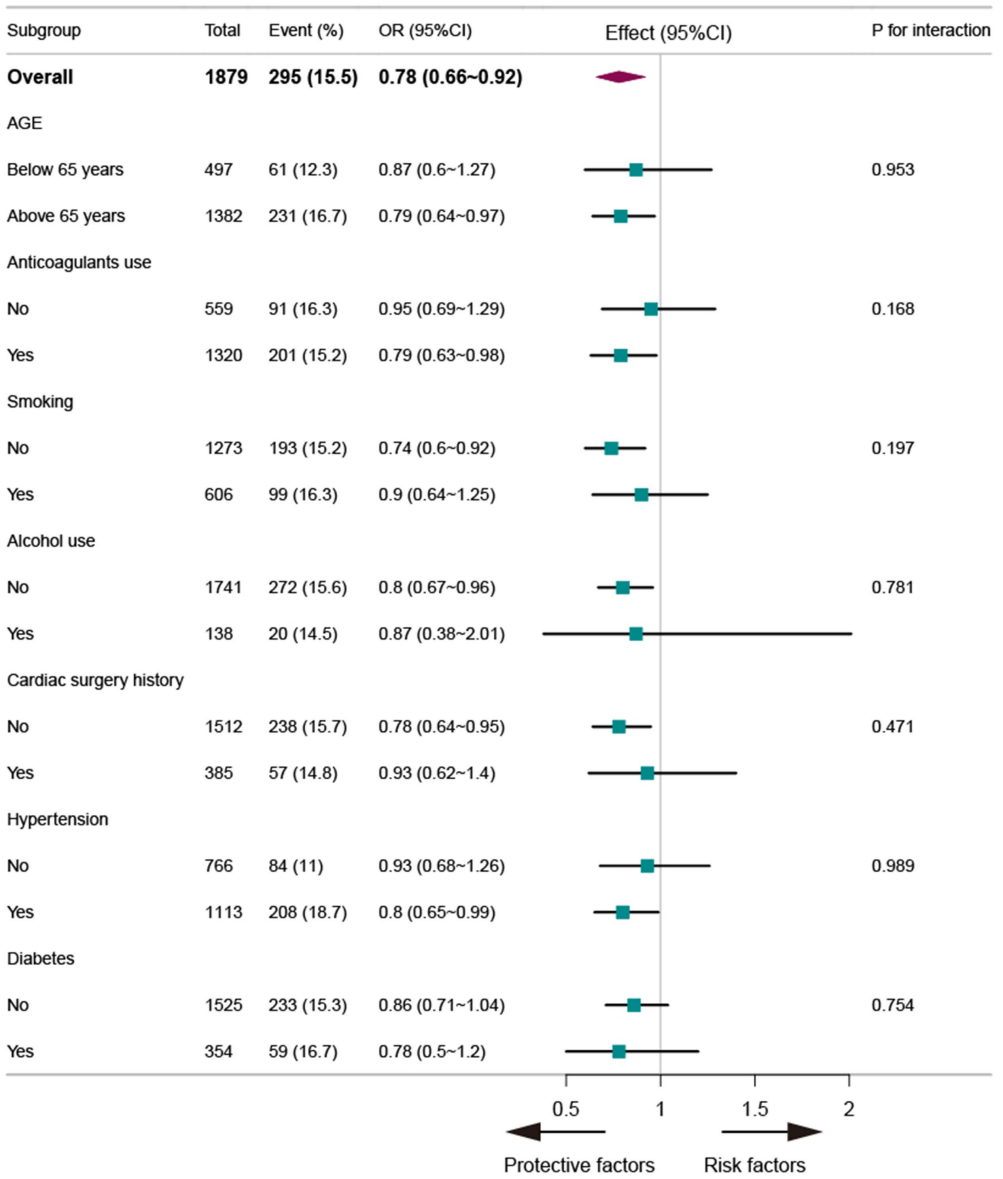
The forest plot between ALI and ischemic stroke in subgroups with AF.

To further investigate the influence of other risk factors on the correlation between ALI and ischemic stroke, subgroup analyses were performed according to the following stratification variables: age, smoking, drinking, use of anticoagulants, hypertension, and diabetes (Figure 2). Subgroup analyses indicated a consistent relationship between ALI and ischemic stroke across all groups, with no significant interactions among the evaluated variables (*p* > 0.05).

### Sensitivity analyses

**Figure 3:**
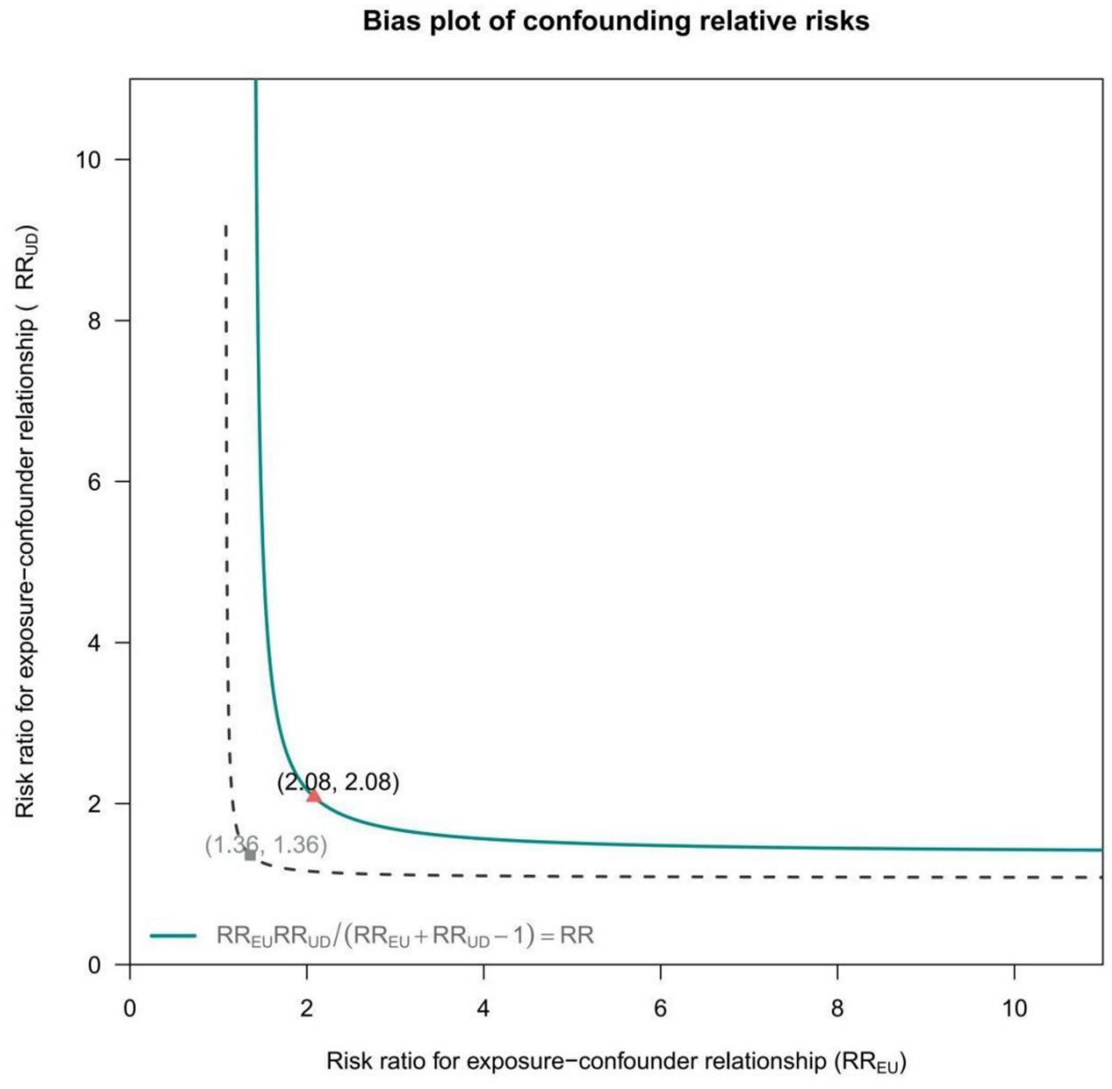
Calculation of unmeasured confounding E-values for ALI grouped ORs.

The results of the sensitivity analyses are shown in the Supplementary material. The matched unadjusted analysis of the primary outcome was not significantly different from that of the fully adjusted model. Additionally, a PSM analysis was conducted to adjust for primary confounding covariates between the ALI groups, further evaluating the robustness of our results. Consistent outcomes were obtained even after accounting for multiple factors. The primary outcomes were similar to those obtained by employing the doubly robust method in the context of IPW. We generated an E-value to assess the reliability of our results in relation to unmeasured confounding factors. Our findings were deemed meaningful unless there was an unmeasured confounding factor that with a greater OR for ischemic stroke higher than 2.08 (Figure 3).

## Discussion

The prognosis in patients with AF and ischemic stroke is often poor, which seriously affects their quality of life (32, 33). Recently, several studies have been conducted on biomarkers for predicting the occurrence of ischemic stroke in patients with AF, including cardiac imaging indicators, electrocardiogram characteristics, atherosclerosis-related markers, circulating biomarkers, and new gene markers (11). Among these, immune-inflammatory indicators are currently a research hotspot. The present study was conducted to explore the correlation and predictive value of a new immune-inflammatory indicator, ALI, with ischemic stroke risk in patients with AF. Previous studies have shown that inflammatory response is closely associated with the occurrence and development of AF. For example, the nucleotide-binding oligomerization domain-like receptor protein 3 (NLRP3) inflammasome is related to the occurrence and development of various types of AF (34), and the anti-inflammatory drug colchicine can be used to treat postoperative and early AF (35). In addition, inflammation is an independent risk factor for thromboembolism and ischemic stroke in patients with AF. Cardioembolic stroke caused by AF results in a stronger immune inflammatory response (36). The mechanism of stroke caused by AF is mainly related to the hypercoagulable state, prethrombotic state, and left atrial thrombus formation in patients with AF (37). Inflammation plays a crucial role in the prethrombotic state and left atrial thrombus formation in patients with AF, which may be related to inflammation-induced endothelial damage and dysfunction, platelet activation, and activation of the coagulation cascade (38). Inflammatory factors, such as CRP, have been shown to be associated with the presence of left atrial thrombus in patients with AF (39). In recent years, some comprehensive indicators that can reflect the body’s inflammatory state have emerged, including the NLR, platelet-to-lymphocyte ratio (PLR), systemic immune-inflammation index, and systemic inflammatory response index (SIRI) (40). Previous studies have shown that NLR and PLR have strong predictive potentials for the prognosis of cardiovascular diseases and related mortality (36). Some studies also revealed that NLR can be used to assess stroke risk and prognosis in patients with AF (41). Another meta-analysis showed that NLR in patients with AF with increased stroke risk was significantly higher than that in patients with AF with reduced stroke risk. When NLR was ≥3, the stroke risk in patients with AF increased by 1.4 times (42). Patients with AF and stroke often in a malnourished state characterized by decreased muscle, fat, and bone mass. Malnutrition impairs the function of the body’s immune system, increases the risk of infection, enhances oxidative stress, and causes free radical accumulation that damages tissues and triggers chronic inflammation. A study has confirmed that severe malnutrition is associated with systemic immune and mitochondrial metabolic disorders, and controlling inflammation and enhancing mitochondrial energy metabolism may help reduce the in-hospital mortality rate of patients with severe malnutrition (43).

Combining inflammatory and nutritional factors, ALI, as a comprehensive indicator for assessing the inflammatory state, can more comprehensively reflect the body’s immune inflammatory state, compared with inflammatory indicators such as NLR, PLR, and SIRI. Currently, there are few studies on ALI in relation to AF combined with ischemic stroke, and it is unclear whether ALI can serve as a predictor of stroke risk in patients with AF. The participants of the present study were divided into two groups according to the optimal cut-off value of ALI (17.2). Patients with AF and low ALI are at high risk of ischemic stroke. The same results were obtained in the propensity score-weighted, multivariable, IPTW, and doubly robust regression analysis models. RCS analyses revealed a linear dose–response relationship between baseline ALI and ischemic stroke risk, indicating that ALI level can serve as an important predictor of ischemic stroke in patients with AF. This result is consistent with previous speculations and may provide a new idea for the early screening of high-risk groups for ischemic stroke among patients with AF. Therefore, ALI, a simple, economical, and easily accessible biomarker, should be further evaluated in clinical practice. This single-center cross-sectional retrospective study may be subject to biases and data incompleteness, necessitating further large-sample studies to confirm the findings regarding the critical value of ALI evaluated in this study.

While our study provides valuable insights into the relationship between ALI and ischemic stroke risk in patients with AF, several critical limitations must be acknowledged to contextualize the findings appropriately. The cross-sectional design, although methodologically appropriate for hypothesis generation, inherently limits causal inference and temporal sequence determination, a fundamental constraint that warrants cautious interpretation of the observed associations. Despite its robust sample size (*n* = 2,630), our single-center cohort may introduce selection bias and limit the generalizability of findings to broader, more diverse populations, particularly those with varying healthcare access and demographic profiles. Future investigations should prioritize prospective, multi-center designs with follow-up periods to validate our findings and elucidate the temporal dynamics of ALI in relation to ischemic stroke.

To our knowledge, limited prior work has addressed to show a significant inverse correlation between ALI and ischemic stroke risk in patients with AF. In this study, higher ALI levels showed an independent linear association with lower ischemic stroke incidence, and may have potential implications for ischemic stroke risk stratification in AF management.

## Data Availability

The original contributions presented in the study are included in the article/Supplementary material, further inquiries can be directed to the corresponding author.
