# Supplementary material for: Association between advanced lung cancer inflammation index levels and ischemic stroke in patients with atrial fibrillation: a propensity score-matching analysis
Source: Front Neurol. 2026 Jan 7;16:1652042. doi: 10.3389/fneur.2025.1652042 (PMC12819322; doi:10.3389/fneur.2025.1652042)
Supplement: Supplementary file 1 [file Data_Sheet_1.pdf]

Table1 : miss data

Variable	Miss. freq	Miss. percentage
Age, years	0	0
albumin, g/L	23	0.87
ALT, U/L	23	0.87
Anticoagulants, n (%)	0	0
anxious, n (%)	0	0
ASTT, U/L	23	0.87
Blood calcium, mol/L	0	0
Blood Chloride, mmol/L	0	0
Blood Potassium, mmol/L	0	0
Blood Sodium, mmol/L	0	0
BMI, kg/m²	681	25.89
BNP, ng/L	124	4.71
ischemic stroke, n (%)	0	0
CK, U/L	23	0.87
CK.MB, U/L	23	0.87
Coronary Heart Disease, n (%)	0	0
Coronary Stent Implantation Surgery, n (%)	0	0
creatinine, μmol/L	10	0.38
CRP, mg/L	109	4.14
D2.polymer, μg/L	126	4.79
Diabetes, n (%)	2	0.08
Diastolic Blood Pressure, mmHg	0	0
drink.alcohol, n (%)	0	0
Ejection Fraction, %	250	9.51
Fasting Blood Glucose, mmol/L	52	1.98
FT3, pmol/L	415	15.78
FT4, pmol/L	411	15.63
Gender, n (%)	0	0
Globulin, g/L	25	0.95
HDL Cholesterol, mmol/L	77	2.93
Heart Rate, bpm	0	0
Heart Valve Disease, n (%)	0	0
Height, m	649	24.68
Hemoglobin, g/L	0	0
Hyperlipidemia, n (%)	0	0
hypertension, n (%)	0	0
INR	55	2.09
Interventricular Septum Thickness, mm	250	9.51
LDH, U/L	23	0.87

LDL Cholesterol, mmol/L	76	2.89
Left Atrium Diameter, mm	249	9.47
Left Ventricle Diameter, mm	249	9.47
Left Ventricular Posterior Wall Thickness, mm	251	9.54
Lymphocyte Count, × 10^9 /L 16	16	0.61
Monocyte Count, × 10^9 /L	19	0.72
Myoglobin, ng/mL	24	0.91
Neutrophil Count, × 10^9 /L	10	0.38
Neutrophil-to-Lymphocyte Ratio,	27	1.03
NYHA Functional Classification, grade	0	0
Pacemaker Implantation Surgery, n (%)	0	0
Platelet Count, × 10^9 /L	0	0
Prothrombin Time, s	55	2.09
Radiofrequency Ablation, n (%)	0	0
Red Cell Distribution Width, %	0	0
Red Blood Cell Count, × 10^{12}/L	0	0
Right Atrium Diameter, mm	249	9.47
Right Ventricle Diameter, mm	249	9.47
Smoking Status, n (%)	0	0
Systolic Blood Pressure, mmHg	0	0
TC, mmol/L	76	2.89
Triglyceride, mmol/L	76	2.89
TSH, mIU/L	355	13.5
Types of Atrial Fibrillation, n (%)	0	0
urea, mmol/L	10	0.38
Uric Acid, µmol/L	10	0.38
White Blood Cell Count, × 10^9 /L	0	0

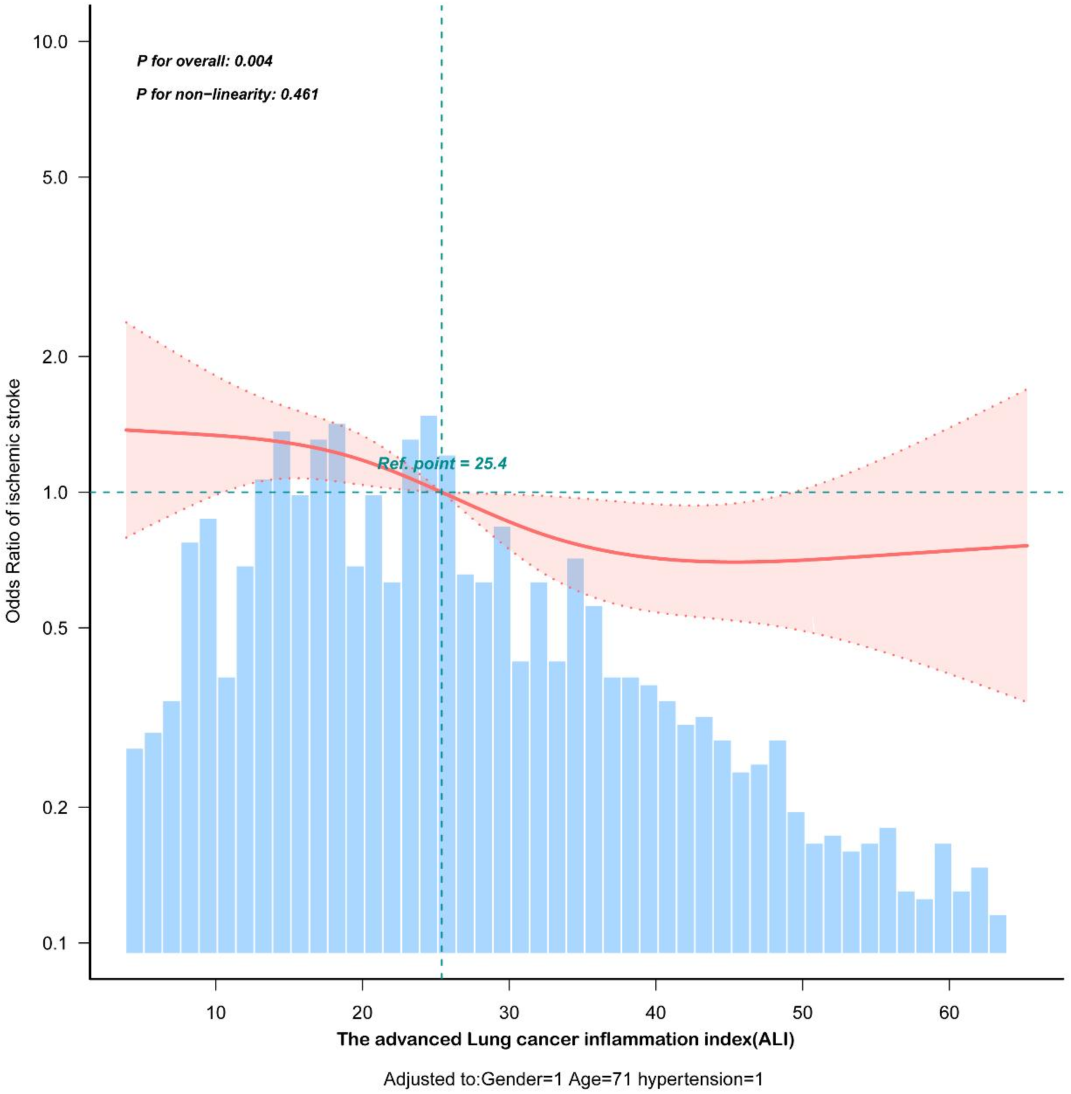

Figure 1. The restricted cubic spline plots for the outcome of ischemic stroke based on varying levels of the ALI. The background histograms (depicted in light blue) represent the percentage density distribution of the ALI within the study population. The heavy central lines indicate the estimated adjusted odds ratios, while the shaded ribbons denote the 95% confidence intervals. The horizontal dotted lines signify an odds ratio of 1.0, designated as the reference point. This reference point is established at the median level of the ALI and also corresponds to the lowest risk for ischemic stroke for each plot (ALI level at 25.4).

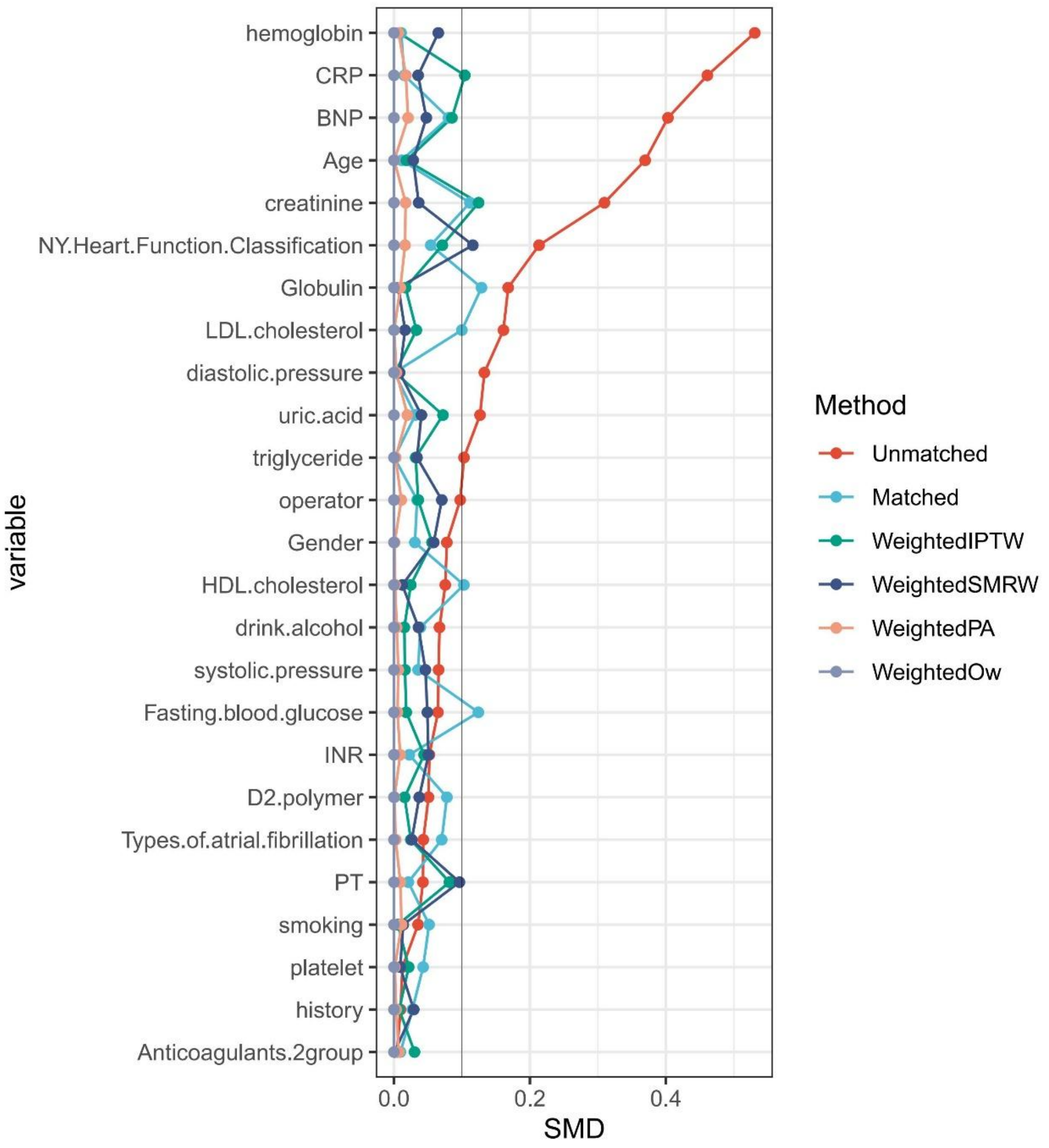

Figure 2. SMD Plot.

Figure 3. PSM ROC.

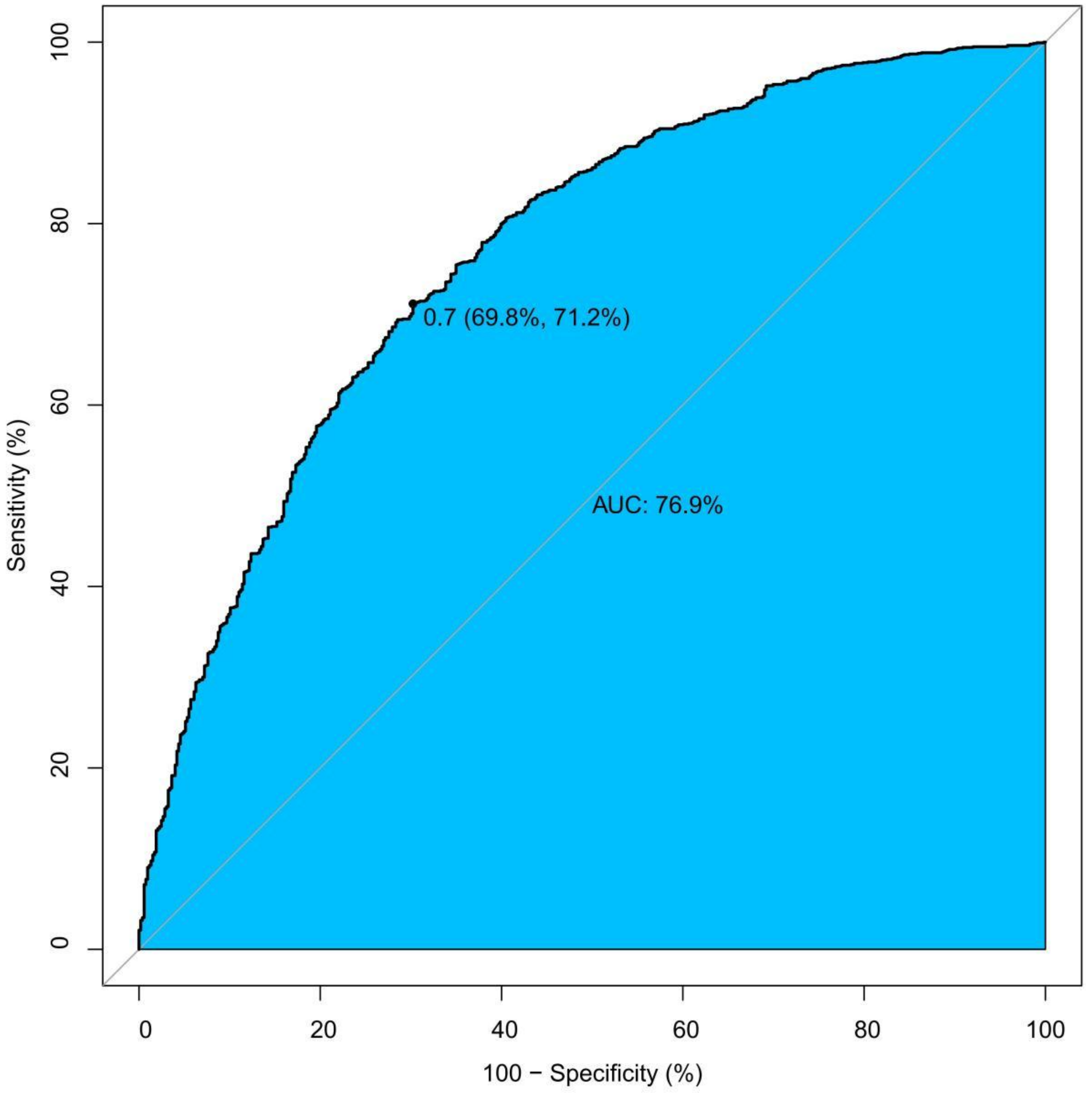

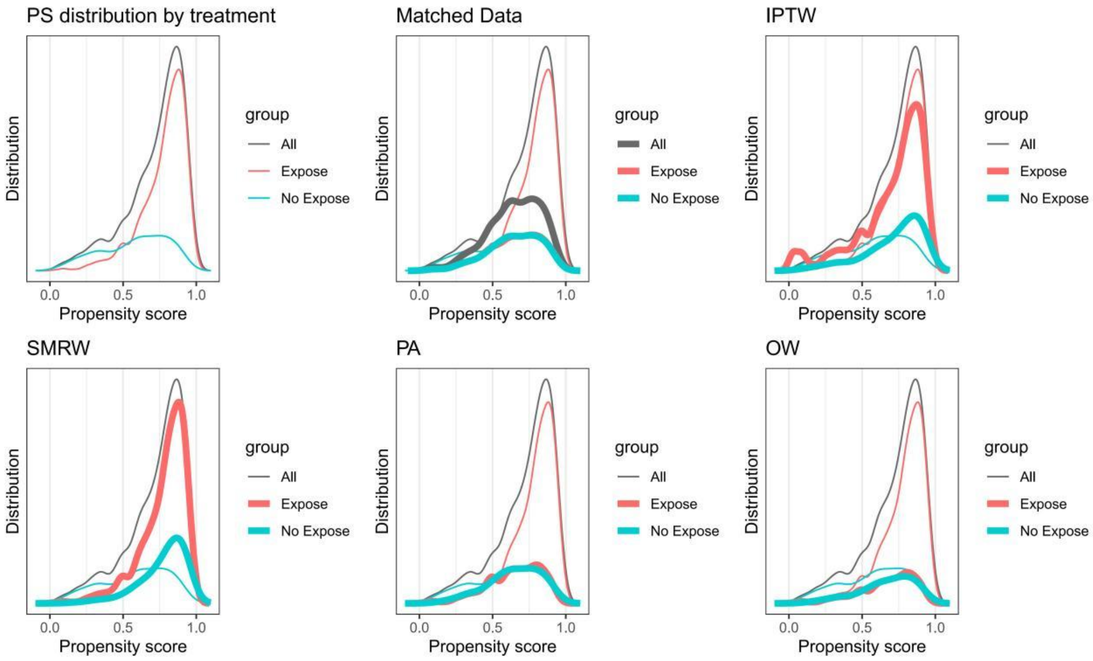

Figure 4 .SMD.plot-2

Table2. Propensity Score for ALI.17.2group

Models	Item1	Variable	OR (95%CI)	P value
Unmatched.crude	ALI.17.2group: 2 vs 1	ALI.17.2group2	0.63 (0.49~0.82)	0.001
Multivariable.adjusted	ALI.17.2group: 2 vs 1	ALI.17.2group2	0.62 (0.46~0.84)	0.002
PropensityScore.adjusted	ALI.17.2group: 2 vs 1	ALI.17.2group2	0.65 (0.48~0.87)	0.004
PropensityScore.Matched	ALI.17.2group: 2 vs 1	ALI.17.2group2	0.66 (0.46~0.94)	0.023
Weighted.IPTW	ALI.17.2group: 2 vs 1	ALI.17.2group2	0.66 (0.51~0.86)	0.002
Weighted.SMRW	ALI.17.2group: 2 vs 1	ALI.17.2group2	0.66 (0.5~0.85)	0.002
Weighted.PA	ALI.17.2group: 2 vs 1	ALI.17.2group2	0.65 (0.45~0.93)	0.017
Weighted.Ow	ALI.17.2group: 2 vs 1	ALI.17.2group2	0.65 (0.42~1)	0.05

Age+Gender+smoking+drink.alcohol+systolic.pressure+diastolic.pressure+heart.rate+hemoglobin+platelet+RDW.SD+red.blood.cell+Blood.potassium+Blood.sodium+Blood.Chlorine+blood.calcium+creatinine+uric.acid+Globulin+Fasting.blood.glucose+triglyceride+HDL.cholesterol+LDL.cholesterol+BNP+CRP+PT+INR+D2.polymer+history+operator+Anticoagulants.2group+Myo; Match.num=1; caliper=0.1.

Table 2.Diagnostic Tests and ROC Analysis with Youden's index

Variable_ROC_Item	Value
ALI.new_threshold	28.7953
ALI.new_specificity	0.4641
ALI.new_sensitivity	0.6758
ALI.new_accuracy	0.4971
ALI.new_tn	736
ALI.new_tp	198
ALI.new_fn	95
ALI.new_fp	850
ALI.new_npv	0.8857
ALI.new_ppv	0.1889
ALI.new_fdr	0.8111
ALI.new_fpr	0.5359
ALI.new_tpr	0.6758
ALI.new_tnr	0.4641
ALI.new_fnr	0.3242
ALI.new_1-specificity	0.5359
ALI.new_1-sensitivity	0.3242
ALI.new_1-accuracy	0.5029
ALI.new_1-npv	0.1143
ALI.new_1-ppv	0.8111
ALI.new_precision	0.1889
ALI.new_recall	0.6758
ALI.new_youden	1.1398
ALI.new_closest.topleft	0.3924

Table 3.The Optimal Cutoff Values Based on Intergroup Comparison with Minimum P-Values

cut1	n	n.per	y	y.per	dump	or	pvalue	p.adjust
14.453125	376/1503	0.20/0.80	79/214	0.21/0.14	b2	0.62	0	1.45
15.95031245	456/1423	0.24/0.76	95/198	0.21/0.14	b2	0.61	0	0.48
14.466073137276	377/1502	0.20/0.80	79/214	0.21/0.14	b2	0.63	0	1.59
14.5438347596644	378/1501	0.20/0.80	79/214	0.21/0.14	b2	0.63	0	1.75
14.5441870441871	379/1500	0.20/0.80	79/214	0.21/0.14	b2	0.63	0	1.92
14.5480643331006	380/1499	0.20/0.80	80/213	0.21/0.14	b2	0.62	0	1.23
14.5508960085867	381/1498	0.20/0.80	80/213	0.21/0.14	b2	0.62	0	1.35
14.5518261526903	382/1497	0.20/0.80	80/213	0.21/0.14	b2	0.63	0	1.49
14.5613618368963	384/1495	0.20/0.80	80/213	0.21/0.14	b2	0.63	0	1.8
14.5858794080836	385/1494	0.20/0.80	80/213	0.21/0.14	b2	0.63	0	1.97
14.5893919094756	386/1493	0.21/0.79	80/213	0.21/0.14	b2	0.64	0	2.16
14.6111763337026	387/1492	0.21/0.79	80/213	0.21/0.14	b2	0.64	0	2.36
14.6273513513514	388/1491	0.21/0.79	80/213	0.21/0.14	b2	0.64	0	2.59
14.6430298146656	389/1490	0.21/0.79	80/213	0.21/0.14	b2	0.64	0	2.83
14.6459430453396	390/1489	0.21/0.79	80/213	0.21/0.14	b2	0.65	0	3.09
14.6731769791083	391/1488	0.21/0.79	80/213	0.20/0.14	b2	0.65	0	3.37
14.7041294167731	392/1487	0.21/0.79	81/212	0.21/0.14	b2	0.64	0	2.21
14.715699613764	393/1486	0.21/0.79	81/212	0.21/0.14	b2	0.64	0	2.42
14.7253261960522	394/1485	0.21/0.79	81/212	0.21/0.14	b2	0.64	0	2.65
14.770290532027	395/1484	0.21/0.79	81/212	0.21/0.14	b2	0.65	0	2.89
14.7860870334507	396/1483	0.21/0.79	82/211	0.21/0.14	b2	0.64	0	1.89
14.8104911671232	397/1482	0.21/0.79	82/211	0.21/0.14	b2	0.64	0	2.07
14.8529081832969	398/1481	0.21/0.79	82/211	0.21/0.14	b2	0.64	0	2.27
14.8566636003003	399/1480	0.21/0.79	82/211	0.21/0.14	b2	0.64	0	2.48
14.881660899654	400/1479	0.21/0.79	82/211	0.20/0.14	b2	0.65	0	2.71
14.9050187826025	401/1478	0.21/0.79	83/210	0.21/0.14	b2	0.63	0	1.77
14.9096433810907	402/1477	0.21/0.79	83/210	0.21/0.14	b2	0.64	0	1.94
14.9124939231891	403/1476	0.21/0.79	84/209	0.21/0.14	b2	0.63	0	1.25
14.9429634198155	404/1475	0.22/0.78	84/209	0.21/0.14	b2	0.63	0	1.37
14.9830531914894	405/1474	0.22/0.78	84/209	0.21/0.14	b2	0.63	0	1.51
14.996790155159	406/1473	0.22/0.78	84/209	0.21/0.14	b2	0.63	0	1.65
15.0883865095334	407/1472	0.22/0.78	85/208	0.21/0.14	b2	0.62	0	1.06
15.1045633064516	408/1471	0.22/0.78	85/208	0.21/0.14	b2	0.63	0	1.17
15.1165133171913	409/1470	0.22/0.78	85/208	0.21/0.14	b2	0.63	0	1.28
15.1407017892653	410/1469	0.22/0.78	86/207	0.21/0.14	b2	0.62	0	0.82
15.1541940789474	411/1468	0.22/0.78	86/207	0.21/0.14	b2	0.62	0	0.9
15.155221714708	412/1467	0.22/0.78	86/207	0.21/0.14	b2	0.62	0	0.99
15.1794358051316	413/1466	0.22/0.78	86/207	0.21/0.14	b2	0.63	0	1.09
15.2094939439087	414/1465	0.22/0.78	86/207	0.21/0.14	b2	0.63	0	1.2
15.2302958280712	415/1464	0.22/0.78	86/207	0.21/0.14	b2	0.63	0	1.32
15.2317274043623	416/1463	0.22/0.78	86/207	0.21/0.14	b2	0.63	0	1.44

15.2358274112934	417/1462	0.22/0.78	87/206	0.21/0.14	b2	0.62	0	0.93
15.2776549976426	418/1461	0.22/0.78	87/206	0.21/0.14	b2	0.62	0	1.02
15.3040811660741	419/1460	0.22/0.78	88/205	0.21/0.14	b2	0.61	0	0.65
15.3200692041522	420/1459	0.22/0.78	88/205	0.21/0.14	b2	0.62	0	0.72
15.3455977138242	421/1458	0.22/0.78	89/204	0.21/0.14	b2	0.61	0	0.45
15.366389896533	423/1456	0.23/0.77	89/204	0.21/0.14	b2	0.61	0	0.55
15.3727587291601	424/1455	0.23/0.77	89/204	0.21/0.14	b2	0.61	0	0.61
15.376218989133	425/1454	0.23/0.77	89/204	0.21/0.14	b2	0.62	0	0.67
15.3779307524537	426/1453	0.23/0.77	89/204	0.21/0.14	b2	0.62	0	0.73
15.4012394366197	427/1452	0.23/0.77	89/204	0.21/0.14	b2	0.62	0	0.81
15.4421534936999	428/1451	0.23/0.77	89/204	0.21/0.14	b2	0.62	0	0.89
15.4423156778916	429/1450	0.23/0.77	89/204	0.21/0.14	b2	0.63	0	0.97
15.4872608500008	430/1449	0.23/0.77	90/203	0.21/0.14	b2	0.62	0	0.62
15.5123019257604	431/1448	0.23/0.77	91/202	0.21/0.14	b2	0.61	0	0.39
15.5386991150442	432/1447	0.23/0.77	91/202	0.21/0.14	b2	0.61	0	0.43
15.5415886377125	433/1446	0.23/0.77	91/202	0.21/0.14	b2	0.61	0	0.48
15.5693281493002	434/1445	0.23/0.77	92/201	0.21/0.14	b2	0.6	0	0.3
15.6202153110048	435/1444	0.23/0.77	92/201	0.21/0.14	b2	0.6	0	0.33
15.6213821427416	436/1443	0.23/0.77	92/201	0.21/0.14	b2	0.61	0	0.37
15.649089390142	437/1442	0.23/0.77	92/201	0.21/0.14	b2	0.61	0	0.4
15.6638958379051	438/1441	0.23/0.77	92/201	0.21/0.14	b2	0.61	0	0.45
15.6813624407464	439/1440	0.23/0.77	92/201	0.21/0.14	b2	0.61	0	0.49
15.681666373751	440/1439	0.23/0.77	93/200	0.21/0.14	b2	0.6	0	0.31
15.6961266916558	441/1438	0.23/0.77	93/200	0.21/0.14	b2	0.6	0	0.34
15.7134016973126	442/1437	0.24/0.76	93/200	0.21/0.14	b2	0.61	0	0.38
15.7152522640059	443/1436	0.24/0.76	93/200	0.21/0.14	b2	0.61	0	0.41
15.7215596010323	444/1435	0.24/0.76	93/200	0.21/0.14	b2	0.61	0	0.46
15.7322448979592	445/1434	0.24/0.76	93/200	0.21/0.14	b2	0.61	0	0.5
15.7366775458709	446/1433	0.24/0.76	94/199	0.21/0.14	b2	0.6	0	0.32
15.7620584751864	447/1432	0.24/0.76	94/199	0.21/0.14	b2	0.61	0	0.35
15.781190433618	448/1431	0.24/0.76	94/199	0.21/0.14	b2	0.61	0	0.39
15.817407663004	449/1430	0.24/0.76	94/199	0.21/0.14	b2	0.61	0	0.42
15.8659638554217	450/1429	0.24/0.76	94/199	0.21/0.14	b2	0.61	0	0.47
15.9122085048011	451/1428	0.24/0.76	94/199	0.21/0.14	b2	0.61	0	0.51
15.9164199937429	452/1427	0.24/0.76	94/199	0.21/0.14	b2	0.62	0	0.57
15.9172422045952	453/1426	0.24/0.76	95/198	0.21/0.14	b2	0.61	0	0.36
15.9406969348746	454/1425	0.24/0.76	95/198	0.21/0.14	b2	0.61	0	0.4
15.9493033021569	455/1424	0.24/0.76	95/198	0.21/0.14	b2	0.61	0	0.44
15.9666611646584	457/1422	0.24/0.76	96/197	0.21/0.14	b2	0.6	0	0.3
16.0871640322796	458/1421	0.24/0.76	96/197	0.21/0.14	b2	0.61	0	0.33
16.1361945890738	459/1420	0.24/0.76	96/197	0.21/0.14	b2	0.61	0	0.37
16.1849767080745	460/1419	0.24/0.76	96/197	0.21/0.14	b2	0.61	0	0.4
16.2039680232716	461/1418	0.25/0.75	96/197	0.21/0.14	b2	0.61	0	0.45
16.2047672253259	462/1417	0.25/0.75	96/197	0.21/0.14	b2	0.62	0	0.49

16.2190118868123	463/1416	0.25/0.75	96/197	0.21/0.14	b2	0.62	0	0.54
16.2362689037199	464/1415	0.25/0.75	96/197	0.21/0.14	b2	0.62	0	0.59
16.2500150133919	466/1413	0.25/0.75	96/197	0.21/0.14	b2	0.62	0	0.71
16.2651869158878	467/1412	0.25/0.75	97/196	0.21/0.14	b2	0.61	0	0.45
16.2902407733389	468/1411	0.25/0.75	97/196	0.21/0.14	b2	0.62	0	0.5
16.3033486276569	469/1410	0.25/0.75	98/195	0.21/0.14	b2	0.61	0	0.32
16.3219448097455	470/1409	0.25/0.75	98/195	0.21/0.14	b2	0.61	0	0.35
16.3573870481928	471/1408	0.25/0.75	99/194	0.21/0.14	b2	0.6	0	0.22
16.3851583710407	472/1407	0.25/0.75	100/193	0.21/0.14	b2	0.59	0	0.14
16.3853453524808	473/1406	0.25/0.75	100/193	0.21/0.14	b2	0.59	0	0.15
16.3854166666667	474/1405	0.25/0.75	100/193	0.21/0.14	b2	0.6	0	0.17
16.3881302427256	475/1404	0.25/0.75	100/193	0.21/0.14	b2	0.6	0	0.18
16.3916039096299	476/1403	0.25/0.75	100/193	0.21/0.14	b2	0.6	0	0.2
16.3962446412455	477/1402	0.25/0.75	101/192	0.21/0.14	b2	0.59	0	0.13
16.4123578678795	478/1401	0.25/0.75	101/192	0.21/0.14	b2	0.59	0	0.14
16.4303782327586	479/1400	0.25/0.75	101/192	0.21/0.14	b2	0.59	0	0.15
16.4600076540375	480/1399	0.26/0.74	101/192	0.21/0.14	b2	0.6	0	0.17
16.4828303850156	481/1398	0.26/0.74	102/191	0.21/0.14	b2	0.59	0	0.11
16.5064018186186	482/1397	0.26/0.74	102/191	0.21/0.14	b2	0.59	0	0.12
16.5287155599905	483/1396	0.26/0.74	102/191	0.21/0.14	b2	0.59	0	0.13
16.5597178864609	484/1395	0.26/0.74	102/191	0.21/0.14	b2	0.59	0	0.14
16.5649670482137	485/1394	0.26/0.74	102/191	0.21/0.14	b2	0.6	0	0.16
16.5790476190476	486/1393	0.26/0.74	102/191	0.21/0.14	b2	0.6	0	0.17
16.5862714850299	487/1392	0.26/0.74	102/191	0.21/0.14	b2	0.6	0	0.19
16.6199474676724	488/1391	0.26/0.74	102/191	0.21/0.14	b2	0.6	0	0.21
16.6831871637544	489/1390	0.26/0.74	102/191	0.21/0.14	b2	0.6	0	0.23
16.6896492207912	490/1389	0.26/0.74	102/191	0.21/0.14	b2	0.61	0	0.26
16.7365959686459	491/1388	0.26/0.74	102/191	0.21/0.14	b2	0.61	0	0.28
16.7927101271388	492/1387	0.26/0.74	102/191	0.21/0.14	b2	0.61	0	0.31
16.8600037232412	493/1386	0.26/0.74	102/191	0.21/0.14	b2	0.61	0	0.34
16.8712357311935	494/1385	0.26/0.74	102/191	0.21/0.14	b2	0.61	0	0.38
16.8876644848259	495/1384	0.26/0.74	103/190	0.21/0.14	b2	0.61	0	0.24
16.9006140342278	496/1383	0.26/0.74	104/189	0.21/0.14	b2	0.6	0	0.15
16.9230630273849	497/1382	0.26/0.74	104/189	0.21/0.14	b2	0.6	0	0.17
16.9489870637051	498/1381	0.27/0.73	104/189	0.21/0.14	b2	0.6	0	0.18
16.9566368366285	499/1380	0.27/0.73	105/188	0.21/0.14	b2	0.59	0	0.11
16.9684559504656	500/1379	0.27/0.73	105/188	0.21/0.14	b2	0.59	0	0.13
16.9692225263993	501/1378	0.27/0.73	105/188	0.21/0.14	b2	0.6	0	0.14
16.9743560432861	502/1377	0.27/0.73	105/188	0.21/0.14	b2	0.6	0	0.15
17.0045222764582	503/1376	0.27/0.73	105/188	0.21/0.14	b2	0.6	0	0.17
17.0221265411183	504/1375	0.27/0.73	105/188	0.21/0.14	b2	0.6	0	0.19
17.0383119248466	505/1374	0.27/0.73	105/188	0.21/0.14	b2	0.6	0	0.2
17.0601200661233	506/1373	0.27/0.73	105/188	0.21/0.14	b2	0.61	0	0.23
17.0653831073242	507/1372	0.27/0.73	105/188	0.21/0.14	b2	0.61	0	0.25

17.0696087795559	508/1371	0.27/0.73	105/188	0.21/0.14	b2	0.61	0	0.27
17.0775149615275	509/1370	0.27/0.73	105/188	0.21/0.14	b2	0.61	0	0.3
17.0868804664723	510/1369	0.27/0.73	105/188	0.21/0.14	b2	0.61	0	0.33
17.0914834258404	511/1368	0.27/0.73	105/188	0.21/0.14	b2	0.62	0	0.36
17.1032764425859	512/1367	0.27/0.73	105/188	0.21/0.14	b2	0.62	0	0.4
17.1272342984857	513/1366	0.27/0.73	105/188	0.20/0.14	b2	0.62	0	0.43
17.1277744871747	514/1365	0.27/0.73	105/188	0.20/0.14	b2	0.62	0	0.47
17.1574068028988	515/1364	0.27/0.73	106/187	0.21/0.14	b2	0.61	0	0.3
17.1887274600721	516/1363	0.27/0.73	106/187	0.21/0.14	b2	0.62	0	0.33
17.2044221049616	517/1362	0.28/0.72	107/186	0.21/0.14	b2	0.61	0	0.11
17.2128478677357	518/1361	0.28/0.72	107/186	0.21/0.14	b2	0.61	0	0.23
17.2919836956522	519/1360	0.28/0.72	107/186	0.21/0.14	b2	0.61	0	0.26
17.379635824389	520/1359	0.28/0.72	107/186	0.21/0.14	b2	0.61	0	0.28
17.3887638350534	521/1358	0.28/0.72	107/186	0.21/0.14	b2	0.61	0	0.31
17.4119982168997	522/1357	0.28/0.72	107/186	0.20/0.14	b2	0.62	0	0.34
17.4134707140412	523/1356	0.28/0.72	107/186	0.20/0.14	b2	0.62	0	0.37
17.4309637939984	524/1355	0.28/0.72	107/186	0.20/0.14	b2	0.62	0	0.41
17.4791173943469	525/1354	0.28/0.72	107/186	0.20/0.14	b2	0.62	0	0.44
17.484703433923	526/1353	0.28/0.72	107/186	0.20/0.14	b2	0.62	0	0.49
17.4867647058824	527/1352	0.28/0.72	107/186	0.20/0.14	b2	0.63	0	0.53
17.4888752493478	528/1351	0.28/0.72	107/186	0.20/0.14	b2	0.63	0	0.58

Table 4 Calculate EVaule of OR

Item	point	lower	upper
RR	1.37	1.08	1.75
E-values	2.08	1.36	NA

Each point along the curve defines a joint relationship between the two sensitivity parameters that could potentially explain away the estimated effect.
